# The Eye in the Sky: Combined Use of Unmanned Aerial Systems and GPS Data Loggers for Ecological Research and Conservation of Small Birds

**DOI:** 10.1371/journal.pone.0050336

**Published:** 2012-12-11

**Authors:** Airam Rodríguez, Juan J. Negro, Mara Mulero, Carlos Rodríguez, Jesús Hernández-Pliego, Javier Bustamante

**Affiliations:** 1 Department of Evolutionary Ecology, Estación Biológica de Doñana CSIC, Seville, Spain; 2 Department of Wetland Ecology, Estación Biológica de Doñana CSIC, Seville, Spain; Universidad Nacional Autonoma de Mexico, Mexico

## Abstract

Technological advances for wildlife monitoring have expanded our ability to study behavior and space use of many species. But biotelemetry is limited by size, weight, data memory and battery power of the attached devices, especially in animals with light body masses, such as the majority of bird species. In this study, we describe the combined use of GPS data logger information obtained from free-ranging birds, and environmental information recorded by unmanned aerial systems (UASs). As a case study, we studied habitat selection of a small raptorial bird, the lesser kestrel *Falco naumanni*, foraging in a highly dynamic landscape. After downloading spatio-temporal information from data loggers attached to the birds, we programmed the UASs to fly and take imagery by means of an onboard digital camera documenting the flight paths of those same birds shortly after their recorded flights. This methodology permitted us to extract environmental information at quasi-real time. We demonstrate that UASs are a useful tool for a wide variety of wildlife studies.

## Introduction

Biotelemetry (or bio-logging science) enables the remote measurement of data pertaining to free-ranging animals using attached electronic devices [1], [2]. These devices are becoming increasingly sophisticated, monitoring behavioral, physiological and even some environmental parameters, and linking them to spatio-temporal movements [3], [4]. As such, biologgers have become a fundamental tool for the development of an emerging discipline called “movement ecology”, aimed at studying all kind of movements by all kind of organisms [5].

Currently, GPS data loggers constitute the lightest devices providing accurate spatio-temporal records, but its use is mainly constrained by the fact that most of them need to be retrieved after deployment to download the data and by battery size (the heaviest part of these devices). Small batteries are exhausted quickly, giving information during a short period of time. Unfortunately, given the relatively heavy mass of some of these devices, high-resolution telemetry still is a technological challenge for field biologists working with small animals [1], [3]. As a rule of thumb in birds, devices should weigh <3–5% of the bird's body mass [6], but the majority of bird species have a body mass lower than 100 g, and the mean mass for 6.000 species is estimated at only 37 g [7]. At present, and with currently available GPS devices weighting several grams, a plethora of studies tracking detailed movements of just large bird species, such as raptors [8], [9] or seabirds [10], are being published. This is seriously skewing our knowledge of movement strategies, and thus home range dimensions as well as total daily distances travelled by non-migratory individuals in the Class Aves.

A new generation of biologgers, known as animal-borne video and environmental data collection systems (AVEDs), have been heralded as the latest revolution in the tracking of wild animals as, in principle, these systems would enable researchers to see what the animal sees in the field [3], [11]. A word of caution has also been raised regarding the cost/benefit ratio of some of these systems, and their applicability (see [11]–[13]). In the case of birds, the species that have carried AVED's for research purposes include large seabirds [14], [15] and crows [16], all of which are well above the mean size in Class Aves [7]. Therefore, the combination of spatio-temporal data with other data provided by biotelemetry (e.g. environmental information) is not feasible for small sized animals [3].

Unmanned aerial systems (UASs) may constitute a useful complement to retrieve environmental data [17], [18], and can be especially interesting for small animals where other techniques involving more weight cannot be applied. Low cost UASs have recently undergone an intense development, leaving the realm of technological wars to become an affordable (Table S1), safe and user-friendly option for a wide variety of wildlife studies [17]–[19].

In this paper, we describe the combined use of GPS data loggers and environmental information recorded by UASs to study habitat selection of a small bird species, the lesser kestrel *Falco naumanni*, living in a highly dynamic landscape. After downloading the spatio-temporal information from the kestrels, we programmed the UASs to fly and document with pictures the paths of those same birds shortly after their flight, extracting environmental information at quasi-real time that we used to study the availability of different habitat types along the bird flightpath. Therefore, obtaining high-resolution images becomes a useful monitoring technique to study habitat selection and/or foraging behavior that can provide invaluable information for conservation and management [20], specially in situations in which foraging decisions may be dependent on structural changes in highly dynamic landscapes.

## Materials and Methods

### Ethics statement

This study has been carried out in accordance with EC Directive 86/609/EEC for animal handling and experiments, and with the current Spanish legislation involving aviation safety. The Regional Government (Junta de Andalucía) approved permits to access to the sampling sites and the animal handling procedures. The Ethics Committee on Animal Experimentation from Doñana Biological Station approved the research plan of HORUS project.

### Study species

Our model species, the lesser kestrel, is one of the smallest European raptors (wing-span 58–72 cm, body mass 120–140 g). It feeds mainly on insects (i.e., grasshoppers, beetles, crickets), but also on small mammals ([21], [22] and references therein). Its population suffered a severe decline (estimated at more than 30% of the world population) during the second half of the 20^th^ century. However, the population has been considered stable for the last two decades, and consequently, it has been recently downlisted from ‘Vulnerable’ to ‘Least Concern’ according to IUCN criteria [20]. Presumably, the main cause of the decline of the lesser kestrel in western Europe was habitat loss and degradation as a result of agriculture intensification [20]. During the chick rearing period, lesser kestrels select field margins and cereal field as foraging areas [23], [24]. In addition, kestrels associate with grain harvesters to catch the arthropods flushed by these machines. One of the most important structural changes associated with agriculture intensification is field enlargement, and consequently, the reduction of field margins [25]. Likewise, the use of machines to harvest cereal fields has reduced the time of harvesting at a locality to just some weeks or days. So, both factors are concurrently limiting kestrel foraging opportunities.

### Study area

Due to the lesser kestrel decline and also for research purposes, several breeding programs have been put in place in Spain in recent years [26]–[28]. One of these reintroductions was carried out in the roof of our own institute (Doñana Biological Station, Seville, Spain), where we conducted this study. In 2008, a hacking program was started releasing to the wild a total of 149 nestlings (51, 58 and 40 in 2008, 2009 and 2010, respectively) originating from a captive breeding program (DEMA, Almendralejo, Spain, www.demaprimilla.org). In addition, injured adult birds (1–4 individuals) were maintained during four breeding seasons (2008–2011) at an external cage (6×2×2 m) to facilitate conspecific attraction at the colony. Breeding pairs established themselves at the colony after the second year (one, three, six and three breeding pairs in 2009, 2010, 2011, 2012, respectively). The colony is formed by two elongated constructions on the roof of a five-floor building. Forty wooden nest boxes with sliding doors to capture the birds at the nests from inside the building are open to the north wall (see Figure S1). Although the colony is located within the urban area of Seville, it is in the northernmost edge of the city facing agricultural fields and the communication ring of the city (highways, railroads, and a high density of powerline corridors). Agricultural fields extend toward the northwest, the nearest ones being no more than 500 m away from the colony.

### Unmanned Aerial Systems (UASs)

The aerial platform was built into a ST-model Easy Fly plane (St-models, China) with a wingspan of 1.96 m and a weight of about 2,000 g (Figure S2). It is propelled using a brushless electrical engine (lithium polymer battery). The UAS was controlled from a ground station using a long-range radio control system. It carried an onboard video camera, a GPS (10 Hz, Mediatek, model FGPMMOPA6B), a data logger with a barometric altitude sensor Eagletree GPS logger V.4 (Eagletree systems, WA, USA), an Ikarus autopilot (Electronica RC, Spain), which provided flight stabilization and On Screen Display (OSD), and a Panasonic Lumix LX-3 digital photo camera 11MP (Osaka, Japan). The camera was integrated in the plane wing aimed to the ground, and was activated using a mechanical servo, set in speed priority mode and in its widest zoom position. The Ikarus OSD provided GPS information about the position, speed, height and course of the aircraft. These data were combined with the video signal from the camera and sent to the ground station in 2,4 GHz. The autopilot provides stabilization of the aircraft, waypoint following capability (including altitude) and an “emergency return home” function. The take-off and landing of the plane is by manual control. The ground station is composed by a monitor, a DVD recorder, the video receiver and the control signal transmitter with their associated antennas. It also includes a Laptop PC to program the autopilot, to store the pictures and data logs, and to decode in-flight telemetry allowing to track the position of the UAS in real time on a Microsoft map (Redmond, WA, USA).

### Experimental procedures

During the 2011 nestling period (June–July), we fitted 5 g GPS data loggers to both members of two breeding pairs of kestrels using Teflon ribbon backpack harnesses (Micro size, TrackPack, Marshall Radio Telemetry, North Salt Lake, Utah, USA). Two *GiPSy2* GPS data loggers (23×15×6 mm, 1.8 g plus 3.2 g battery, Technosmart, Italy) were programmed in continuous mode (1 fix/sec) for a four hour period. To avoid monitoring abnormal behavior due to capture stress and harness fitting, birds were first captured and fitted with a harness and a 5 g dummy GPS data logger. One week latter birds were recaptured and the dummy substituted by a real GPS data logger programmed to start recording data the next day after recapture. To download the data from the data loggers, birds were recaptured at their nest boxes when they were delivering food to their nestlings, after batteries were exhausted one day latter.

After the download of the bird tracks, six flights were made by the UAS. Three of them with the aim of repeating the flights made by the lesser kestrels from their nests to their foraging areas, and three additional flights following random transects over the agricultural fields. Random flights connected locations randomly selected in a straight line. Pictures of the area overflown were taken using the onboard photo camera that was shooting continuously while the aircraft was following the routes.

### Data analyses

Given that the accuracy on altitude measurements of the GPS used for navigation is relatively low, to georeference the pictures taken by the camera onboard we used information provided by an Eagletree GPS logger V.4 (Eagletree systems, WA, USA) that includes a barometric altitude sensor. The pictures were georeferenced using a customized extension of ENVI software that used Eagletree data to generate GeoTIFF files.

**Figure 1:**
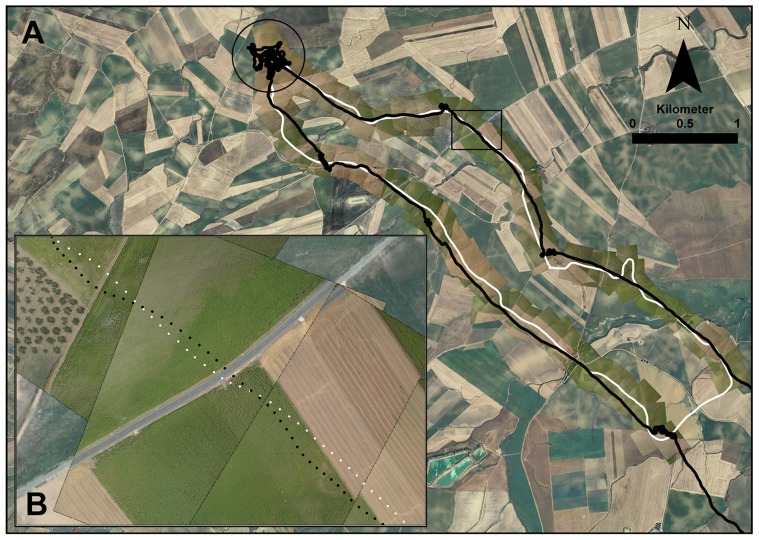
Track of a lesser kestrel foraging flight over the images obtained by an unmanned aerial system. **A** White and black tracks correspond to unmanned aerial system and lesser kestrel flights, respectively. The circle indicates the hunting area. The rectangle indicates the enlarged area in B. **B** High resolution images showing sunflowers, olive trees, road and harvested cereal fields.

Images taken from the UAS let us clearly identify six types of field crops (or land uses): harvested cereal, fully grown cereal (unharvested), olive trees, sunflowers, fallow land and ‘others’ (e.g., farm houses, barns, roads, streams). Using ArcGIS v.10 (ESRI, Redlands, CA, USA), we measured the percentage of total distance overflown by the UAS over each field type, as well as the number of field margins crossed by the UAS. To evaluate the capacity of UAS to follow kestrels' routes, we used the tool ‘NEAR’ implemented in ArcGIS to calculate the distance between each kestrel fix to the nearest UAS fix. For this analysis, we deleted the part of kestrel tracks related to active hunting activities and distinct from displacement flights between the colony and the actual foraging grounds (easily recognizable by changes in elevation, direction and speed between consecutive fixes at the distal part of tracks; see Figure 1).

## Results

**Figure 2:**
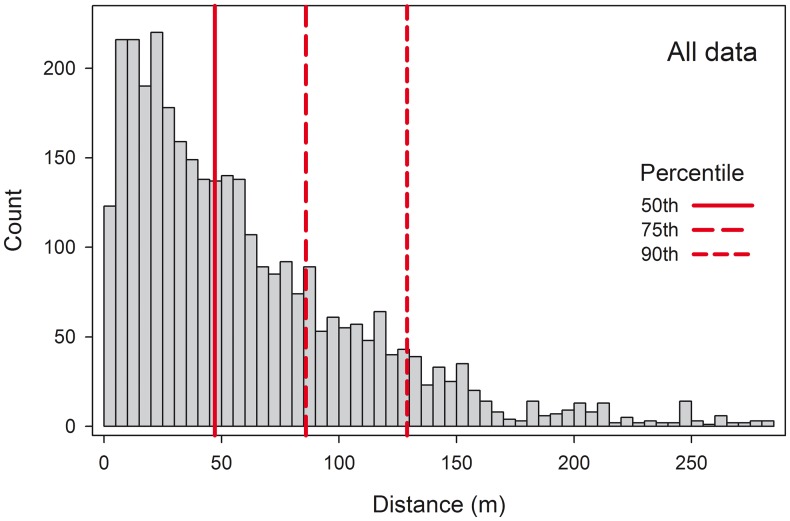
Distribution of nearest distances between kestrel and UAS fixes. Fixes from each flight are combined. Fixes were taken one per second.

We obtained 4,460 high resolution images along six different flights (three following the kestrels plus three random transects), but there was a high degree of overlap, and we finally selected 466 of them to build the photo-mosaics. The kestrel actual flights recorded by the bird data loggers were always included in the imagery taken by the UAS (Figure 1). UASs followed the kestrel tracks with high precision, with the majority of recorded distances between kestrel and UAS fixes lower than 50 m. The 75^th^ and 90^th^ percentiles were 85.9 and 128.9 m, respectively (Figure 2). Spatial resolution of imagery depends on the altitude at which images are taken (Figure S3). Our UAS flew at a mean altitude of 184 m, and thus, the mean spatial resolution of imagery was 7.7 cm.

The area overflown by kestrels is intensively cultivated, being divided into small plots of sunflower, cereal (mainly wheat), olive groves, and other minor cultivations. Proportions of overflown field types did not show significant differences between flights (i.e. go, return and random transect flights; Table 1), so that kestrels flew them in proportion to their availability. Additionally, go and return flights did not differ from the random flights performed by the UASs in relation to the proportion of habitat types. This suggests that the kestrels did not follow specific prospecting strategies when getting to the foraging areas or leaving them. However, local environmental conditions affecting kestrel flight decisions at a microscale, such as wind gusts, could not be recorded in our aerial photographs.

**Table 1:** Characteristics of the areas overflown by the UAS during the simulated lesser kestrel flights (go and return) and random transects.

	Go flight	Return flight	Random transects	Kruskal-Wallis test	*P*
Harvested cereal (%)	37.7±6.4	32.2±16.5	32.5±8.4	3.31	0.19
Cereal (%)	9.6±5.5	7.4±5.9	5.2±3.9	1.86	0.39
Olive trees (%)	2.6±2.4	2.8±2.5	3.9±3.5	0.62	0.73
Sunflowers (%)	44.6±7.2	48.4±11.5	53.4±1.9	1.42	0.49
Fallow lands (%)	2.1±0.8	3.2±4.3	0.5±0.9	1.19	0.55
Others (%)	3.4±0.6	6.1±4.5	4.5±3.4	0.80	0.67
N of margins per Km	6.8±1.2	6.0±0.2	6.6±1.4	0.62	0.73
Mean flight length (Km)	6.97±1.27	7.12±0.63	6.35±1.36	1.06	0.58

## Discussion

The lesser kestrel is one of the smallest raptors in Eurasia and its size, and particularly body mass, poses a serious limit to the weight of biotelemetry devices or loggers that can be attached (about 5–6 g maximum, depending on the individual) to record spatial position or behavioral activity. During the course of our investigations on the lesser kestrel, that began in 1988 [29], we have always pursued to get an accurate knowledge of their daily movements at their breeding grounds. Applying radio transmitters and direct behavioral observations of unmarked individuals we have been able to determine foraging habitat preferences [30]–[32], but soon realized that we lost track of the birds more often than we located them, biasing our studies to locations near the breeding colony. Later on, geolocators have permitted us to determine that kestrels from southern Spain wintered in the Sahel area of western Africa [33]. While this was a breakthrough with conservation implications, due to the low spatial precision of the technology, it was useless to monitor movements at the breeding grounds. It was not until recently that programmable GPS data loggers small enough to be fitted in a lesser kestrel became available. This technology has revealed that individual kestrel sometimes forage 15–20 km away in straight line from the breeding colony (data not shown). A question emerged as what type of habitats the kestrels were selecting out of the available ones. Lesser kestrels are colonial birds that exploit sudden outburst of invertebrate prey [34]. They defend no foraging grounds and flocks of several birds may be sighted hovering and diving at times on ground-based or low flying potential prey [34]. Although information on crop types may be obtained from satellite images, kestrels are known to respond to rapid structural changes of vegetation in their environment [32]. A flock of kestrels may hunt on a particular harvested field for one or two days and never be back. Keeping this in mind, we used the UAS, as it could be deployed immediately after we downloaded GPS data from individual kestrels.

The results presented here are meant as a demonstration of the capabilities of the UAS to obtain a mosaic of images corresponding to the actual full foraging trips of free-ranging small birds. The UAS flight paths reproduced the kestrel flights reliably, as indicated by the fact that their trajectories tended to be less than 100 m apart (see Figure 2). The precision fit of the UAS autopilot depends on the number of waypoints included in the settings (note that our Ikarus autopilot admits 32 waypoints), as well as the meteorological conditions, so we foresee precision will be improved using better autopilots. In addition, images taken by the camera installed in the UAS flying at average altitude of 184 m above sea level covered an area on the ground that always contained the bird track projection (Figure S4). Post-processing of the pictures resulted in a mosaic of georeferenced images allowing an evaluation of habitat types as well as plot sizes and other landscape features, such as grassy field margins, roads, power lines, or even the presence of harvesters in the fields (data not shown; but see Figure 1 for examples of field margins and roads). In fact, UAS images taken from a mean altitude of 184 m showed a higher resolution (7.7 cm) than freely available satellite images (e.g. those coming from MODIS, 250 m, or Landsat TM or ETM+, 30 m), under request commercial satellite images (e.g. DigitalGlobe, Colorado, USA, 30–65 cm) or orthophotographies (e.g. Junta de Andalucía, Spain, 1–1.5 m).

To obtain habitat information, there are other alternative (or complementary) options (see Table S2). The most basic would be to get to the study area and survey it by foot or using a ground vehicle. This is time consuming, it has logistical complications and some landscape variables (at large scales) may not be easily quantified. Stationary cameras or sensors scattered in the landscape can provide interesting information about environmental changes, but they involve a huge economic investment and previous knowledge of animal movements, long post-processing of the data, and it is always risky for the equipment, especially in open areas where they can be damaged or stolen. Satellite images are very useful for spatial studies, but their spatial and temporal resolution may not suit research objectives. In our study case, freely available satellite images do not reach the necessary spatial and temporal resolution to distinguish changes in the highly dynamic habitat (e.g. harvested vs. non-harvested fields). For example: NASA's Earth Observing System Data and Information System (EOSDIS) can provide only 250-m resolution images from MODIS sensor twice a day for Spain; but they are affected by clouds and have a spatial resolution too low for our aims. Commercial satellite images with the appropriate spatial resolution could be available, but at a high cost and there is greater delay in data acquisition compared with UAS. Aerial photographs can be ordered from specialized firms, but a mosaic of georeferenced images of the landscape would be quite expensive, and it would be logistically problematic to obtain the pictures when needed, i.e. at the desired temporal resolution.

In the case of small birds, the recreation of flight paths of birds has been achieved using radio-tracking devices and miniaturized video cameras [11]–[16]. However, if home range is large enough to lose the radio signal or there is no previous information on where the birds are moving, this methodology may bias the results (see [13]). In larger birds, cameras have been attached on them (e.g. seabirds [14], [15]), but in a non-systematic way and with no possibility to get zenithal images of enough high quality that could be processed in a statistical manner. In our case, there is admittedly a delay of several hours between the flight of the bird and that of the UAS, but this is of little relevance for answering most of our ecological questions.

In our study, GPS data for bird positions was obtained at a frequency of one fix-per-second. In the trade-off among fix frequency vs. length of the registration period, we favored the former for improved spatio-temporal accuracy. Our decision rested on two facts: one, this configuration let us to distinguish among soaring, gliding and hunting flights (i.e. hovering and strikes) according to elevation, direction and speed of fixes; and two, the kestrels we were tracking, even if free-ranging, were easily captured in the colony situated on the roof of our headquarters. This condition, the easy of retrieving the GPS data logger to download data, is not met in a majority of investigations on wild birds [13]. Therefore, future technological advances to finely track a wider range of small sized species should include remote wireless downloading of the GPS information by GSM, Bluetooth or radio. For the moment, this technology has only been incorporated to relatively large devices that can only be mounted on correspondingly large bird species (see www.celltracktech.com, www.technosmart.eu, [35]). In addition, UASs can be configured to carry on board additional sensors, such as barometers, thermometers or video cameras. These capabilities of the UAS as a non-intrusive tool for ecological research can also be envisaged as extremely useful in studies of flight dynamics (e.g. recording atmospheric parameters such as temperature, wind direction and strength, or barometric pressure [8]), predator-prey interactions (e.g. recording UV light from prey urine tracks which may attract to predators [36]), social dynamics (e.g. monitoring birds of different species during migration [37]) or behavioral decisions related to the conservation of species (e.g. recording what shearwater fledglings would see when they are fatally attracted to artificial lights during their first flights from nest-burrows to sea [38], [39]). As a future refinement, UASs may also be used to locate and track at a safe distance animals equipped themselves with radio transmitters or other locating devices. All the heavy equipment, such as video or still cameras, would go in the UAS and the animal would just carry a light weight location device.

Our UAS flew programmed routes, providing georeferenced images of the area overflown by kestrels. The combination of the GPS position provided by the data loggers and the images provided by the UAS recreate the trajectory of a bird carrying a camera. It improves, however, the performance of the other techniques available to date to study the environment as conventional fieldwork, satellite imagery, aerial pictures or stationary cameras.

## Supporting Information

Figure S1 **Lesser kestrel breeding colony located at the headquearters of Doñana Biological Station (Seville, Spain).** A) Lesser kestrel colony located at the roof of the headquarters of Doñana Biological Station in Seville. B) Nestlings in the proximity of releasing nest-boxes. C) Fledglings perched in one of the antennas of the building. D) First breeding attempt as seen from the inside of the colony structure. E) Cage with adult birds inside and fledglings resting outside.
(TIF)

Figure S2 **Unmanned Aerial System equipment and operation.** A) Aerial platform. B) Ground station. C) Antennas of control signal transmitters. D) Manual take off.
(TIF)

Figure S3 **Relationship between image resolution and altitude.** Dashed lines indicate the mean altitude flow (184 m) and the mean spatial resolution of the imagery (7.7 cm).
(TIF)

Figure S4 **Distribution of nearest distances between kestrel and UAS fixes.** Fixes were taken one per second.
(TIF)

Table S1 **Budgetary cost of the equipment used in this study.**
(PDF)

Table S2 **Pros and cons of commonly used techniques for recording environmental information.** This table is based on our study case, i.e. an actual case to study the habitat selection of Lesser Kestrel using the kestrel flight tracks. Note that advantages/disadvantages may change according to the aims of the studies.
(PDF)
