# Supplementary figures and images for: The Eye in the Sky: Combined Use of Unmanned Aerial Systems and GPS Data Loggers for Ecological Research and Conservation of Small Birds
Source: PLoS One. 2012 Dec 11;7(12):e50336. doi: 10.1371/journal.pone.0050336 (PMC3519840; doi:10.1371/journal.pone.0050336)

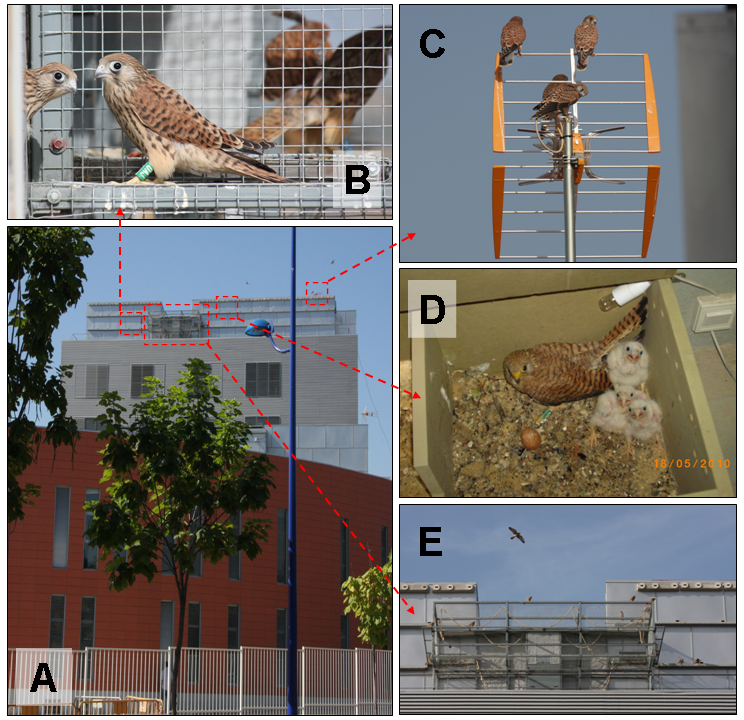

Supplement: Figure S1 — Lesser kestrel breeding colony located at the headquearters of Doñana Biological Station (Seville, Spain). A) Lesser kestrel colony located at the roof of the headquarters of Doñana Biological Station in Seville. B) Nestlings in the proximity of releasing nest-boxes. C) Fledglings perched in one of the antennas of the building. D) First breeding attempt as seen from the inside of the colony structure. E) Cage with adult birds inside and fledglings resting outside. (TIF) [file pone.0050336.s001.tif]

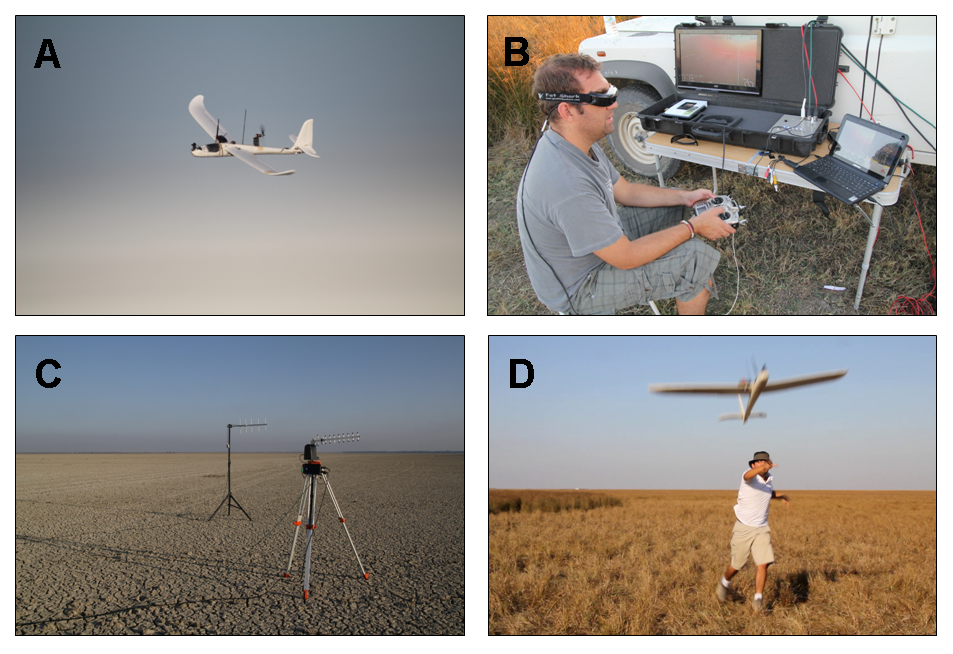

Supplement: Figure S2 — Unmanned Aerial System equipment and operation. A) Aerial platform. B) Ground station. C) Antennas of control signal transmitters. D) Manual take off. (TIF) [file pone.0050336.s002.tif]

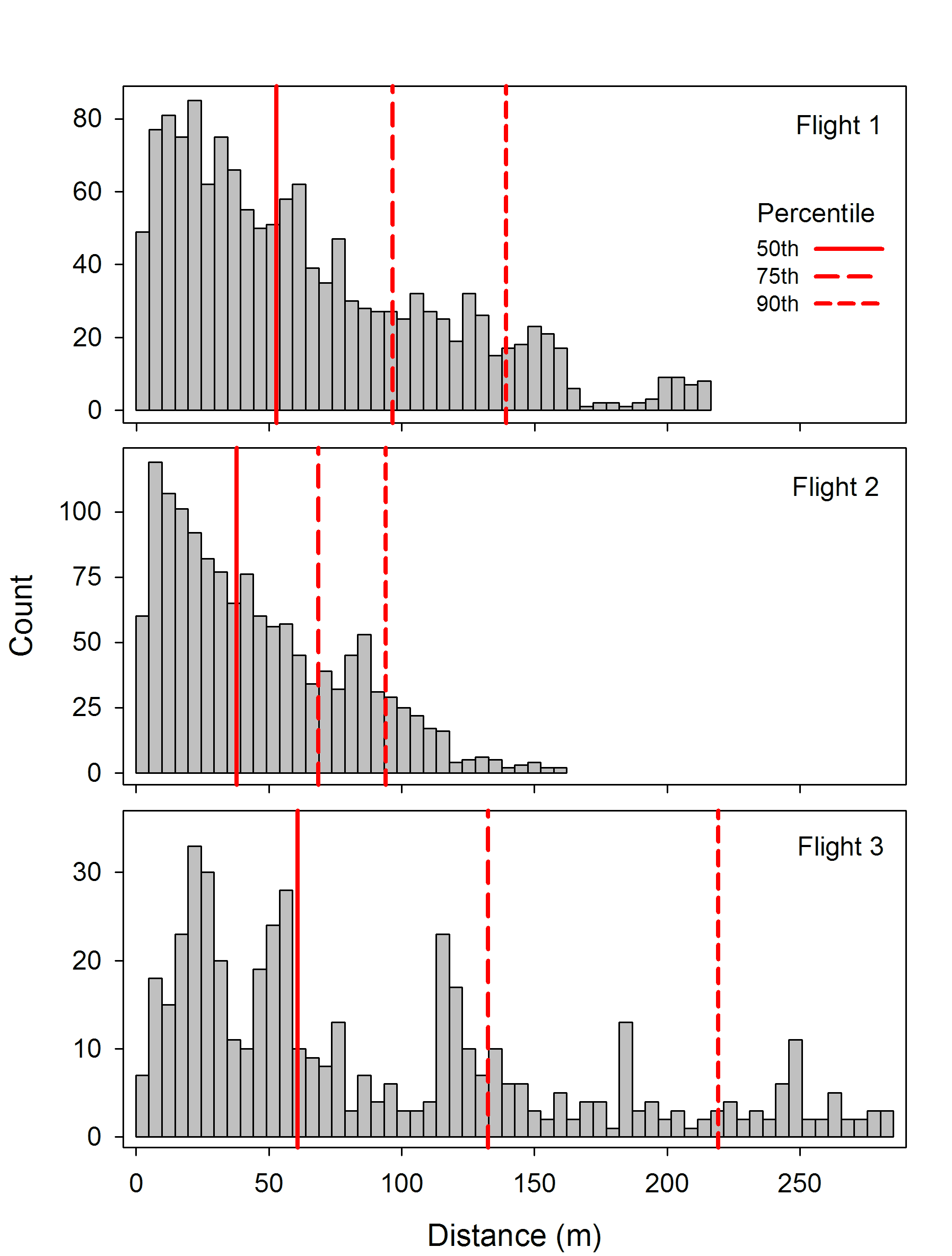

Supplement: Figure S4 — Distribution of nearest distances between kestrel and UAS fixes. Fixes were taken one per second. (TIF) [file pone.0050336.s004.tif]
