# Supplementary material for: The Eye in the Sky: Combined Use of Unmanned Aerial Systems and GPS Data Loggers for Ecological Research and Conservation of Small Birds
Source: PLoS One. 2012 Dec 11;7(12):e50336. doi: 10.1371/journal.pone.0050336 (PMC3519840; doi:10.1371/journal.pone.0050336)
Supplement: Table S1 — Budgetary cost of the equipment used in this study. (PDF) [file pone.0050336.s005.pdf]

Table S1. Budgetary cost of the equipment used in this study.

Component	Price * (€)
Teflon ribbon back-pack	30
GPS data logger	800
Aerial platform (including electronics onboard, GPS, autopilot, stabilization system, FPV camera, Eagle tree with barometric sensors)	1000
Payload	
Panasonic Lumix LX3	450
Antennas and tripods	
Video	1500
Control	400
Ground Control Station (including laptop, monitor and connecting wires)	1500
Total	5680

* Prices paid in Spain in 2011, in Euros. It may vary depending on location.
