# Supplementary material for: The Eye in the Sky: Combined Use of Unmanned Aerial Systems and GPS Data Loggers for Ecological Research and Conservation of Small Birds
Source: PLoS One. 2012 Dec 11;7(12):e50336. doi: 10.1371/journal.pone.0050336 (PMC3519840; doi:10.1371/journal.pone.0050336)
Supplement: Table S2 — Pros and cons of commonly used techniques for recording environmental information. This table is based on our study case, i.e. an actual case to study the habitat selection of Lesser Kestrel using the kestrel flight tracks. Note that advantages/disadvantages may change according to the aims of the studies. (PDF) [file pone.0050336.s006.pdf]

Table S2. Pros and cons of commonly used techniques for recording environmental information. This table is based on our study case, i.e. an actual case to study the habitat selection of Lesser Kestrel using the kestrel flight tracks. Note that advantages/disadvantages may change according to the aims of the studies.

Approach	Advantages	Disadvantages
Survey by foot, on horse back or using a terrestrial vehicle	Low cost of technology.	Economic costs per ha can be high depending on technician salaries. Time consuming. Difficulty to access to some areas (e.g. fenced private farms, rugged or remote areas with no paths/roads). Lack of aerial perspective.
UASs images	High resolution (depending on flying altitude). Versatility to record other variables (e.g. by using thermal, infrared or UV light cameras, or even other sensors such as barometers or thermometers). Possibility to simulate the bird field of vision. Quasi-immediate data collection.	Medium economic costs (see Table S1). Range (depending on UAS characteristics). Limited by favorable weather conditions.
Animal-borne video	Real time.	Device mass is too large to be used in a majority of bird species. Photographed areas are not taken in a systematic way (e.g. no zenithal images).
Commercial satellite images	High resolution (30-65 cm). Versatility to record other variables (e.g. using the 8-band multispectral imagery).	High economic costs. Time lag to set an order (i.e. no immediate data collection).
Medium resolution satellites	Low cost. MODIS and Landsat TM and ETM+ images can be obtained for free.	Low spatial resolution. Optical satellite sensors are limited by clouds this can limit acquisition of simultaneous images
Commercial aerial photography taken from conventional aircraft	High resolution (depending on flying altitude).	High economic costs. Time lag to set an order (i.e. no immediate data collection).
Airborne hyperspectral sensors	High spatial and spectral resolution allows measuring a wide range of environmental variables.	Very high economic cost. Limited availability of sensors and operators. Difficulty of organizing an airborne campaign at short notice.
